# Effects of Vigiis 101-LAB on a healthy population's gut microflora, peristalsis, immunity, and anti-oxidative capacity: A randomized, double-blind, placebo-controlled clinical study

**DOI:** 10.1016/j.heliyon.2020.e04979

**Published:** 2020-09-22

**Authors:** Chien-Li Chen, Jyh-Ming Liou, Tsong-Ming Lu, Yi-Hsien Lin, Chin-Kun Wang, Tzu-Ming Pan

**Affiliations:** a Department of Research and Development Division, SunWay Biotech Co., Ltd., Taipei, Taiwan; b Division of Gastroenterology and Hepatology, Department of Internal Medicine, National Taiwan University Hospital, College of Medicine, National Taiwan University, Taipei, Taiwan; c Department of Neurology, Chung Shan Medical University Hospital, Taichung, Taiwan; d Division of Radiotherapy, Cheng Hsin General Hospital, Taipei, Taiwan; e School of Nutrition, Chung Shan Medical University, Taichung, Taiwan; f Department of Biochemical Science and Technology, College of Life Science, National Taiwan University, Taipei, Taiwan

**Keywords:** Microbiology, Food microbiology, Nutrition, Evidence-based medicine, Gut microflora, Immunity, Anti-oxidative capacity, *Lactobacillus paracasei* subsp. *paracasei* NTU 101, Probiotics

## Abstract

Vigiis 101-LAB capsules are produced from the fermentation of *Lactobacillus paracasei* subsp. *paracasei* NTU 101. We tested effects of Vigiis 101-LAB capsules I or II (5 or 10 billion CFU/day, respectively) on gut microflora in clinical trial I, and on peristalsis, immunity, and anti-oxidative capacity in clinical trial II, during a 4-week randomized, double-blind, placebo-controlled, adaptive-design study. In trial I, 36 subjects were divided into capsule I and placebo groups. After 4 weeks, *Bifidobacterium* spp. and *Lactobacillus* spp. counts were significantly higher in the feces of treatment subjects, with increases of 4.01- and 4.25-fold, respectively. In trial II, 52 subjects were divided into capsule II and placebo groups. After 4 weeks, the treatment group was found to have improved motility, decreased food transit time, and significantly increased immunoglobulin G, immunoglobulin M, and antioxidant activity. Thus, daily administration of Vigiis 101 capsule II can improve peristalsis, immunity, and anti-oxidative capacity.

## 1 Introduction

Probiotic bacteria have become increasingly popular over the last two decades as a result of the expanding scientific evidence indicating their beneficial effects on human health (Kechagia et al., 2013). The internationally accepted definition for probiotics was proposed as any living microbe that, when consumed by humans or other animals, can modify the gut microbial balance leading to a beneficial effect on the host (Havenaar & Huis in't Veld, 1992). Both single and mixed strains can be considered as probiotics, as well as specific combinations of two organisms that enhance each other's growth (probiosis). This synergy improves the gut microflora balance, enhances the immune system, and promotes digestive and nutritionally related biochemical functions (Hooper et al., 2012; Perdigón et al., 1999). Conversely, numerous studies have demonstrated that gut microflora is closely associated with cancers of the digestive system (Rafter, 2004). Alterations in the gut microflora are thought to affect tumor development because harmful microorganisms may enhance tumor formation by inducing procarcinogens. On the other hand, *Lactobacillus* can compete with pathogenic bacteria for the intestinal environment and nutrients, preventing the colonization of pathogenic bacteria that cause infection (Ouwehand et al., 2000). *Lactobacillus* can also produce organic acids, to decrease the pH value of the intestinal tract, and short-chain fatty acids, hydrogen peroxide, bacteriocin, and other antibacterial substances that inhibit the growth of pathogenic bacteria (Naidu et al., 1999). A previous study showed that fermented milk containing probiotics decreased harmful bacteria in feces, such as *Escherichia coli*, while simultaneously increasing *Bifidobacterium* levels in the gut. These findings demonstrate that probiotic supplementation can improve gut microflora (Ayebo et al., 1980). The cell walls of bacteria and some plants can absorb toxins in the gut. Food typically contains some mutagens such as heterocyclic amines that are a by-product of cooking protein-rich foods. Many studies have shown that *Lactobacillus* can adsorb these substances and decrease their mutagenicity (Piotrowska, 2014)**.** These heterocyclic amines could be bound by bacteria, improving host immunity (Orrhage et al., 1994). Another study found that *Bifidobacterium infantis* can stimulate host immune responses and result in tumor inhibition or regression (Sekine et al., 1985). Additionally, the cell walls of probiotic bacteria contain lipopolysaccharide and peptidoglycan, which can activate the host immune system by stimulating CD14 and promote the release of cytokines through signal transduction pathways. This mechanism regulates immune responses and inhibits tumor formation (Pool-Zobel et al., 1996). Long-term administration of probiotics as dietary supplements in daily life can effectively prevent gastrointestinal infections and treat gastrointestinal diseases (Pant et al., 2007; Ouwehand et al., 2002; Saavedra et al., 1994).

As 75% of immune tissues are concentrated near the gut, these tissues are essential for physical health. *Lactobacillus* can maintain the gut ecosystem, preserve gut epithelial integrity, and form a protective barrier, thereby reducing the occurrence of allergies and autoinflammation. Additionally, *Lactobacillus* can increase the expression levels of interferon-α and interferon-γ and induce T-helper 1 (Th1) immune responses, which decrease allergic responses (Chen et al., 1999). Other studies showed that the addition of probiotics to milk whey decreases food allergies, inhibits allergic rhinitis and nasal allergies in infants, and regulates serum IgE levels (Halpern et al., 1991; Van de Water, Keen & Gershwin, 1999).

*Lactobacillus paracasei* subsp. *paracasei* NTU 101 strain (NTU 101) is a local *Lactobacillus* strain that was isolated and selected from the guts of newborns in Taiwan and shows good adaptability, gastric acid resistance, and bile salt resistance characteristics (Lin et al., 2004). This strain can survive for long periods in the human gut, inhibit the growth of harmful bacteria, and play multiple other roles (Tsai et al., 2008). Studies have shown that the NTU 101 strain, or its fermented products, contribute to several functions, namely: (1) improving the function of gut microflora by preventing harmful bacteria from colonizing the gut, thereby exerting protective effects (Tsai et al., 2010); (2) regulating the immune system by stimulating cell activation and proliferation and increasing the secretion of cytokines and antibodies to enhance innate and acquired immune responses (Tsai et al., 2008); (3) repairing damage to the gastric mucosa by inhibiting acute gastric mucosal injury caused by pylorus ligation and acidified alcohol, decreasing lipid peroxide concentrations in the blood and gastric mucosa, increasing the activity of superoxide dismutase, and promoting the synthesis of prostaglandin E2, which protects the mucosa (Liu et al., 2009); and (4) reducing the tendency to synthesize body fat. Indeed, previous studies have shown that both the NTU 101 powder and soymilk fermented with it could inhibit the proliferation and differentiation of adipocyte precursor cells and promote lipolysis by mature adipocytes (Lee et al., 2013). Studies of the regulation of gastrointestinal function showed that NTU 101 could prevent infection by enteric pathogens (Tsai et al., 2010), increase the expression levels of tight junction proteins, and maintain the integrity of intestinal walls (Hung et al., 2016). For metabolic functions, NTU 101 can regulate blood pressure (Liu et al., 2011), blood lipids (Chiu et al., 2006; Tsai et al., 2009; Tsai et al., 2012), blood glucose (Hung et al., 2016), and cholesterol to alleviate metabolic abnormalities. Additionally, NTU 101 also has (6) osteoprotective effects (Chiang and Pan, 2011), (7) protective effects on teeth (Lin and Pan, 2014; Liu et al., 2018), and (8) antioxidant (Liu and Pan, 2010) and inflammation regulatory effects (Chiang et al., 2011).

This study is divided into two sections and focuses on *Lactobacillus* supplementation effects on healthy people using NTU 101. Clinical trials were conducted to assess the effects of the Vigiis 101-LAB capsule I on improving gastrointestinal function and regulating gut flora (clinical trial I), and the Vigiis 101-LAB capsule II on the improvement of peristalsis, immunity, and anti-oxidative capacity (clinical trial II). It was hypothesized that the test product would confer a significant digestive benefit on healthy participants without causing harm, as evaluated using these criteria.

## 2 Materials and methods

### 2.1 Materials

The strain used in the current study was *L. paracasei* subsp. *paracasei* NTU 101 (lyophilized powdered, Vigiis 101-LAB; probiotic powder from SunWay Biotech Co., Ltd., Taipei, Taiwan). The Vigiis 101-LAB mixed lactose, crystalline cellulose, and excipient were made into capsules (Vigiis 101-LAB capsule I; 100 packages; lot number 160501) containing 5 billion bacteria per capsule for the gut flora clinical trial I. The Vigiis 101-LAB mixed lactose, crystalline cellulose, and excipient were also mixed into capsules (Vigiis 101-LAB capsule II; 100 packages; lot number 170320) containing 10 billion bacteria per capsule for clinical trial II on peristalsis, immunity, and anti-oxidative capacity. Maltodextrin was used as a placebo.

### 2.2 Study population

Clinical trials I and II were registered at ClinicalTrials.gov with registration numbers NCT04046432 and NCT04088474. In total, 90 healthy patients meeting the inclusion criteria were assigned randomly to either the Vigiis 101-LAB or the placebo group (Clinical trials I (n = 18) and II (n = 27) each). The gaussian distribution was used in this study. It was assumed that during any measurement values will follow a normal distribution with an equal number of measurements above and below the mean value. All of the subjects completed the trial.

#### 2.2.1 Randomized, double-blind clinical criteria for effects of Vigiis 101-LAB capsule I on gut flora (clinical trial I)

Clinical trial I was conducted from May 2016 to May 2017 at Cheng Hsin General Hospital after acquiring ethics approval from the Institutional Review Board (IRB) (IRB proof document CHGH-IRB No: 538 105B–07 New Case). Subjects voluntarily agreed to participate in the clinical trial and signed a written informed consent form. Inclusion criteria were as follows: (1) healthy adults aged ≧ 20 years and ≦ 65 years, (2) with healthy weight (body mass index: 18.5–24.0), and (3) with no gastrointestinal diseases or current use of medication. Exclusion criteria were as follows: (1) those with a confirmed diagnosis of major illness/injury by a doctor, (2) pregnant women or women planning to get pregnant within half a year, (3) patients with abnormal liver function (aspartate aminotransferase or alanine aminotransferase greater than 2-fold the upper limit of normal), (4) patients with abnormal kidney function (serum creatinine >1.5 mg/dL), (5) patients with abnormal gastrointestinal function (surgery, frequent diarrhea), (6) patients taking drugs that may affect carbohydrate or lipid metabolism, such as female hormones, steroids, blood lipid-lowering drugs, diuretics, or other blood glucose-lowering drugs, and (7) patients who suffered from severe comorbidities during the previous 6 months, including stroke, myocardial infarction, major trauma, or surgery. A randomized, double-blind design was adopted in this study and patients were divided into 2 groups: the test and placebo groups. The Vigiis 101-LAB capsule I was administered orally once per day, one capsule each time. The entire study took 4 weeks, and subjects were prohibited from eating fermented food products, such as miso, kimchi, fermented dairy products, oligosaccharide-containing foods, and lactic acid bacteria-containing products. Subjects also avoided consuming excessive gas-producing foods (such as soybeans and sweet potatoes) during their daily meals and avoided foods that can cause abdominal distension or promote peristalsis (such as lactic acid beverages and oligosaccharide-containing beverages).

After the trial started, subjects recorded their daily number of bowel movements and completed relevant questionnaires at weeks 0, 2, and 4. The subjects visited the doctor once every 2 weeks for monitoring of gastrointestinal function and physiological characteristics. At weeks 0, 1, 2, 3, and 4, fecal samples from the subjects (approximately 0.1–0.5 g) were collected and put into bottles containing an anaerobic diluent and shaken to uniformly mix the feces and diluent. The samples in the bottles were then immediately used for *Bifidobacterium* spp. counts. The collected samples were serially diluted before inoculation into Bifidobacterium iodoacetate medium 25 (BIM-25) and cultured under anaerobic conditions at 37 °C for 3 days before colony enumeration. To enumerate *Lactobacillus* spp., the collected samples were serially diluted in MRS agar and cultured under anaerobic conditions at 37 °C for 3 days. To enumerate *Clostridium perfringens*, the collected samples were serially diluted before inoculation into tryptose sulfite cycloserine (TSC) agar with D-cycloserine and/or TSC agar with egg yolk and cultured under anaerobic conditions at 37 °C for 3 days before colony enumeration. To enumerate coliforms, the collected samples were serially diluted before inoculation into chromogenic *E. coli*/coliform agar and cultured under aerobic conditions at 37 °C for 1 day.

#### 2.2.2 Randomized, double-blind clinical criteria for effects of Vigiis 101-LAB capsule II on peristalsis, immunity, and anti-oxidative capacity (clinical trial II)

Clinical trial II was conducted from March 2017 to April 2018 at Chung Shan Medical University after acquiring ethics approval from the IRB of Taichung Chung Shan Medical University Hospital (IRB proof document CHMUH No: CS17018), and all subjects signed an informed consent form. Inclusion criteria were as follows: (1) healthy adults aged ≧ 20 years and ≦ 65 years, (2) with a healthy weight (body mass index: 18.5–24), (3) and no gastrointestinal diseases or current use of medication. The exclusion criteria were as follows: (1) pregnant or lactating women, (2) subjects who are allergic to *Lactobacillus*, (3) subjects with chronic gastrointestinal diseases, (4) subjects who previously underwent gastrectomy or gastric bypass, (5) subjects with liver, kidney, or heart disease, alcoholism, or uncontrolled diabetes, (6) subjects who had experienced a stroke, psychiatric diseases, or depression in the previous year, and (7) subjects taking drugs that could regulate gastrointestinal function, functional foods, bacteriostatic drugs or supplements, antibiotics, antioxidants, or other unknown drugs within the last 2 weeks. The Vigiis 101-LAB capsule II was administered orally once per day, one capsule each time. Every day, the diet of the subjects was recorded in detail from the pre-stability to post-stability period. Day 1 of week 0 was considered the start of the stability period. Subjects who fulfilled the enrollment criteria were confirmed and not allowed to consume any nutritional supplements while conforming to the national balanced diet recommendations once the test started. During week 0 of the observation period, subjects filled in the dietary records and began administration of the test product on day 1 of week 1 after blood was drawn. Blood was drawn at weeks 4 and 6, and subjects stopped administering the product after blood was drawn on week 4. The trial ended at week 6 after blood was drawn.

### 2.3 Methods

#### 2.3.1 Randomization, treatment, and follow-up

**Figure 1:**
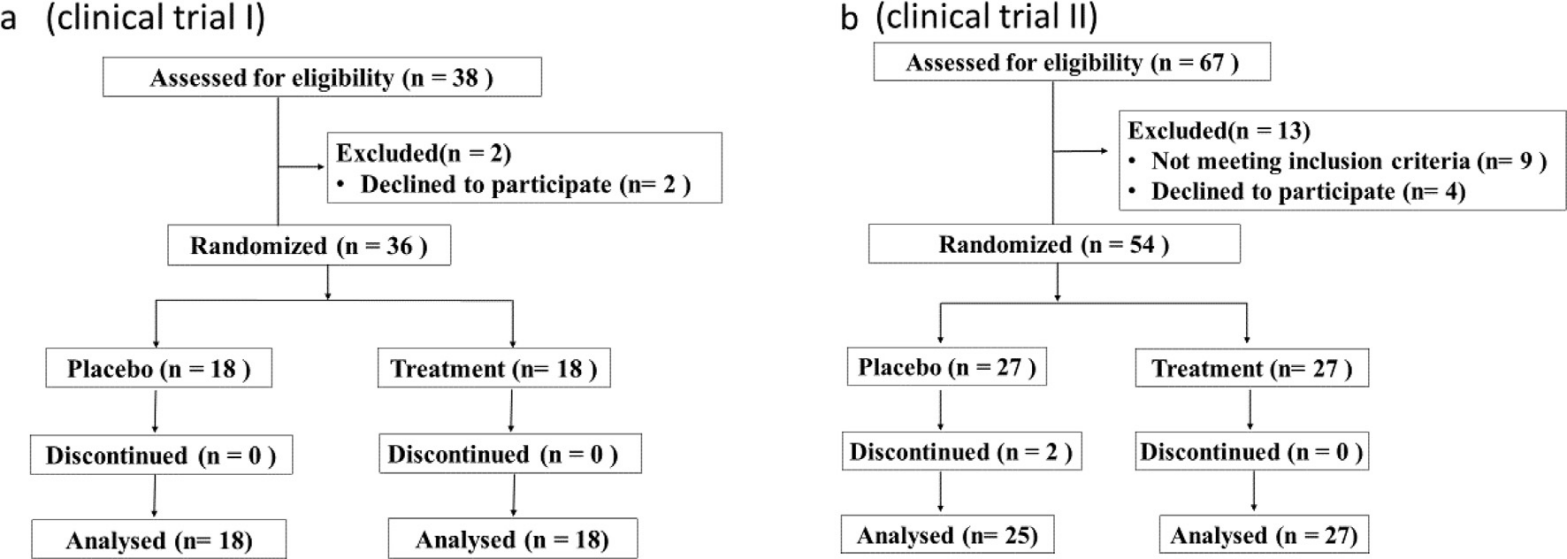
a) CONSORT flow diagram of healthy patients (clinical trial I). b) CONSORT flow diagram of healthy patients (clinical trial II).

Regardless of the study group assignment, eligible patients were randomly assigned to be administered either Vigiis 101-LAB capsule I or II (treatment group) or the placebo (control group) at a ratio of 1:1 during the last visit of the parental study or as soon as possible after that. Randomization was centrally performed using an interactive voice or Web response system. Treatments were stratified based on the study group assignment in the parent trial, and the placebo schedule was based on the drug dose frequency in the parent trial (Figure 1a, b).

#### 2.3.2 Outcome measurements

In clinical trial I primary endpoint of this study was gut microflora. The clinical trial II primary endpoint of this study was intestinal peristalsis. Fecal moisture content and pH value testing: the stool samples were collected once at weeks 0, 1, 2, 3 and 4 in a small box and placed in an anaerobiosis system in clinical trial I. Testing of the small intestine emptying rate: during the stability period before the subject administered the product and after the subject administered the product for 4 continuous weeks, the subjects consumed 120 mL of barium sulfate for small intestine imaging, and fluoroscopy was carried out once every 30 min until the barium was completely expelled from the ileocecal valve. The time required for barium to pass through the ileocecal valve was recorded. Anthropometric measurements included the body height (BH), body weight (BW), blood pressure (BP), midarm circumference (MAC), triceps skinfold (TSF), waist circumference (WC), rump circumference (RC), and heart rate (HR). The body mass index (= BW/BH^2^, kg/m^2^) of the subjects was calculated after measuring their BH and BW. The MAC, WC, and RC were measured using a measuring tape by the same staff member. After obtaining the WC and RC of the subjects, the waist to hip ratio (WC/RC) was calculated. For blood pressure measurements, the systolic blood pressure (SBP) and diastolic blood pressure (DBP) of the right arm were measured. MAC was measured using the same pair of calipers by the same trained staff member. Fasting blood samples were collected for biochemical tests at weeks 0, 4, and 6. The test items included blood biochemistry tests, plasma antioxidant activity (Trolox equivalent antioxidant capacity, TEAC), thiobarbituric acid reactive substances (TBARS), glutathione levels (GSH), glutathione peroxidase (GSH Px), glutathione reductase (GSH Rd), full blood count, and blood electrolytes. Blood biochemical tests were carried out in the laboratory. Dietary assessment for the 24-h diet recall method was used to calculate the average daily intake, which was compared to the actual calories required. Examination of clinical symptoms: improvements in reduced appetite, other changes in appetite, food intake, body weight, hemoglobin, and other markers were observed. To evaluate improvements in poor digestion and absorption, changes in appetite, food intake, gastrointestinal distension, fecal shape, number of bowel movements, gastrointestinal movements, intestinal absorption, and other markers were observed. The designated study endpoint of both trials was the incidence of adverse events. Additional safety endpoints included severe adverse events, adverse events leading to the discontinuation of the study health food (for patients in the Vigiis 101-LAB capsule I group or II group), and abnormalities in creatine kinase levels, liver or kidney function, and electrolyte balance. A prespecified exploratory outcome was defined as the incidence of confirmed cardiovascular events throughout the study.

### 2.4 Sample size and statistical analysis

In this study the sample size was based on a two-sample t-test at a significance level of 0.05 with a two-sided alternative. To achieve randomization, a list of random numbers was used to allocate the two treatments (A, treatment; B, placebo). The clinician on site made this allocation. The person generating the randomized list also did the labeling of the treatments and kept the randomized list. Each numbered supply of the relevant treatment (treatment or placebo) was labeled with that particular number. When allocated, the participant ID number was added to the label details on the capsule containers. Data were expressed as the mean ± standard deviation (SD). The statistical significance of the biochemical analyses was determined by one-way analysis of variance (ANOVA) using the general linear model procedure of the SPSS software (SPSS Institute, Inc., Chicago, IL, USA). This was followed by ANOVA with a paired *t*-test to evaluate the difference before and after test product and placebo administration, while the Student's *t*-test was used to compare the differences between test and placebo groups (*P* ≤ 0.05).

## 3 Results

### 3.1 Effects of Vigiis 101-LAB capsule I on anthropometric measurements and *Bifidobacteria, Lactobacillus*, *Clostridium perfringens,* and coliform counts in the gut (clinical trial I)

**Figure 2:**
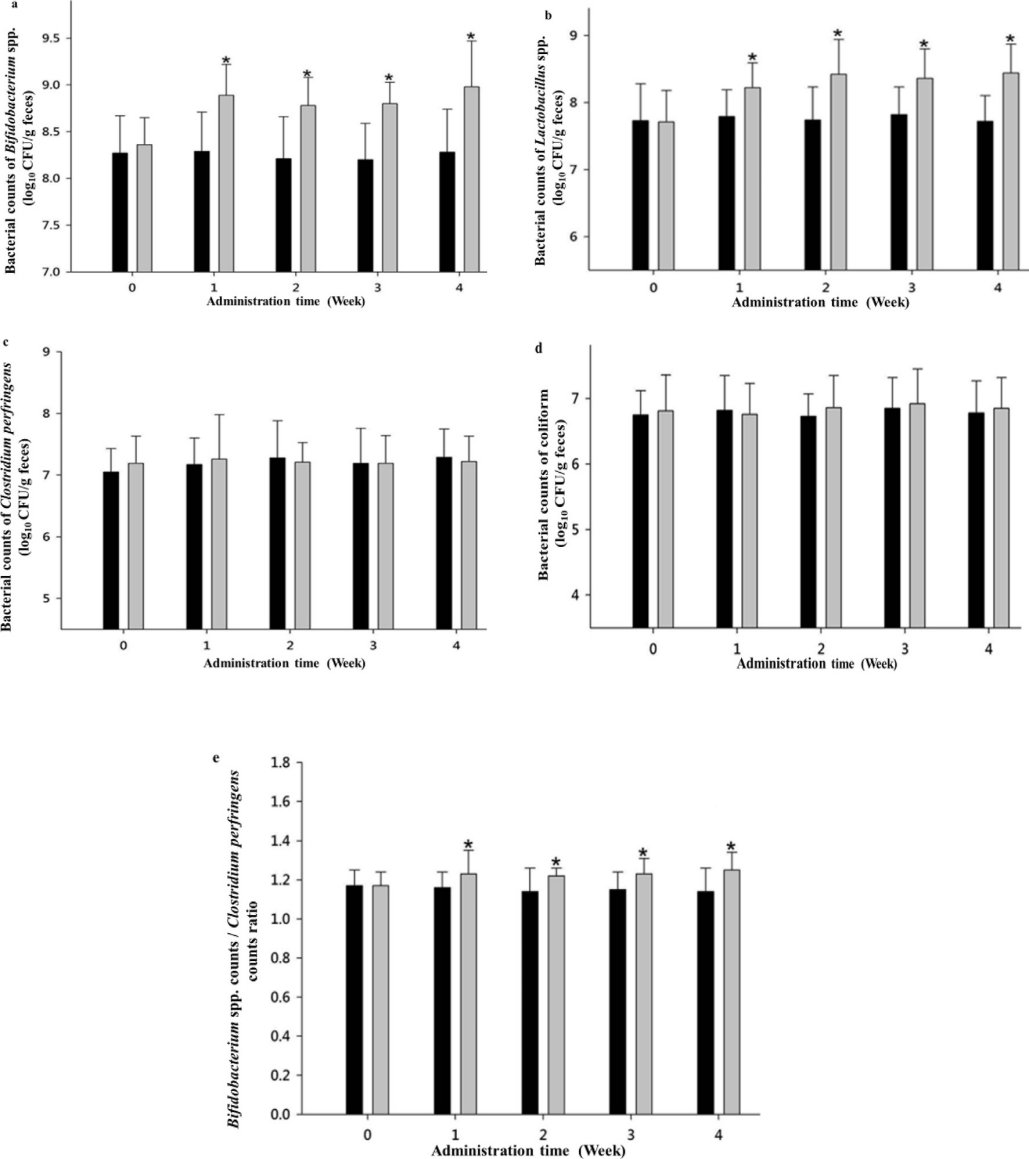
a) Effect of the test product on *Bifidobacterium* spp. counts in human feces. b) Effect of the test product on *Lactobacillus* spp. counts in human feces. c) Effect of the test product on *Clostridium perfringens* counts in human feces. d) Effect of the test product on coliform counts in human feces. e) Effect of the test product on *Bifidobacterium* spp. counts/*Clostridium perfringens* counts ratio in human feces.  Treatment group: Administered a capsule containing 50 mg Vigiis 101-LAB powder;  Placebo group: Administered a capsule without 50 mg Vigiis 101-LAB powder. All data are expressed as mean ± SD, n = 18. The between-group comparison was carried out with a Student's t-test, wherein *P* ≤ 0.05 indicates a significant difference between the treatment and placebo groups at the same time point (marked with an ∗).

The gaussian distribution was used in this study with the assumption that during any measurement values would follow a normal distribution with an equal number of measurements above and below the mean value. In total, 36 subjects were enrolled in this study, with 18 subjects in the treatment group with a mean age of 28.6 years, and 18 subjects in the placebo group with a mean age of 28.3 years (Table 1). Fecal samples were homogenized and diluted before inoculation into the BIM-25 medium. The results (Table 1 and Figure 2a) showed that after 1 week of administration of the test product, the bifidobacterial count in the feces from the test group was significantly higher than that in the placebo group (*P* ≤ 0.05) and bifidobacterial counts at weeks 1–4 in the feces from the test group were 3.72–5.01-fold those of the placebo group. The results in Table 1 and Figure 2b show that after 1 week of administration of the test product, the *Lactobacillus* count in the feces from the test group was significantly higher than that in the feces from patients in the placebo group (*P* ≤ 0.05), and *Lactobacillus* counts at weeks 1–4 in the feces from the test group were 2.69–5.01-fold those of the placebo group. Results shown in Figure 2c demonstrate that after 4 weeks of test product administration, there were no significant differences in the *C. perfringens* count in the feces from patients of the test and placebo groups. Figure 2d shows that after 4 weeks of test product administration, there were no significant differences in coliform counts in the feces from subjects belonging to the test and placebo groups.

**Table 1:** Effect of Vigiis 101-LAB capsule I on anthropometric measurements and beneficial intestinal bacteria of healthy adults (clinical trial I).

	Treatment group			Placebo group		
Age (y)	28.6 ± 4.2			28.3 ± 8.0		
Male	8			9		
Female	10			9		

Time (Week)	*Bifidobacterium* spp. counts (log10 CFU/g)		*Lactobacillus* spp. counts (log10 CFU/g)		*Bifidobacterium* spp./*Clostridium perfringens* ratio	
	Placebo group	Treatment group	Placebo group	Treatment group	Placebo group	Treatment group

0	8.27 ± 0.40	8.36 ± 0.29	7.73 ± 0.55	7.71 ± 0.47	1.17 ± 0.08	1.17 ± 0.07
1	8.29 ± 0.42	8.89 ± 0.33∗	7.79 ± 0.40	8.22 ± 0.37∗	1.16 ± 0.08	1.23 ± 0.12∗
2	8.21 ± 0.45	8.78 ± 0.30∗	7.74 ± 0.49	8.42 ± 0.52∗	1.14 ± 0.12	1.22 ± 0.04∗
3	8.20 ± 0.39	8.80 ± 0.23∗	7.82 ± 0.41	8.36 ± 0.44∗	1.15 ± 0.09	1.23 ± 0.08∗
4	8.28 ± 0.46	8.98 ± 0.49∗	7.72 ± 0.38	8.44 ± 0.43∗	1.14 ± 0.12	1.25 ± 0.09∗

Values are mean ± SD (n = 18). ∗ Significantly different from the initial value (P < 0.05).

### 3.2 Effects of Vigiis 101-LAB capsule I on the ratio of probiotic bacteria and harmful bacteria in the gut (clinical trial I)

After subjects began administration of the Vigiis 101-LAB capsule I or the placebo, the ratios of *Bifidobacterium* to *C. perfringens* counts in the feces of the subjects were compared. Table 1 and Figure 2e show that the ratio of *Bifidobacterium* counts to *C. perfringens* counts were significantly higher in the test group than in the placebo group after 1 week of test product administration. Thus, the Vigiis 101-LAB capsule I increased the number of *Bifidobacteria* counts in the gut and improved the ratio of gut probiotics counts to harmful bacteria counts, allowing *Bifidobacteria* to become the dominant bacterial flora in the gut.

### 3.3 Safety monitoring of Vigiis 101-LAB capsule I subjects (clinical trial I)

During the 4-week trial, the dietary adjustments, consumption of the test product, bowel movements, disease, and discomfort of subjects were recorded every day. During the trial period, the clinician carried out consultations once every 2 weeks and conducted relevant questionnaires to record the dietary and defecation habits of the subjects. No subjects experienced abnormal reactions during the trial.

### 3.4 Effects of Vigiis 101-LAB capsule II on gut microflora, peristalsis, and anthropometric measurements (clinical trial II)

Table 2 shows that a total of 52 subjects were enrolled in this study, with 27 subjects in the treatment group with a mean age of 25.5 years; 25 subjects were in the placebo group with a mean age of 26.0 years. This clinical trial also demonstrates that the anthropometric measurements, including BH, BMI, WC, RC, MAC, TSF, BP, and HR, showed no significant differences between the test and placebo groups (Table 2).

**Table 2:** Effect of chronic administration of Vigiis 101-LAB capsule II on anthropometric measurements of subjects (clinical trial II).

		Treatment group			Placebo group		
		Initial (0 wk)	Administration (4 wk)	Follow-up (6 wk)	Initial (0 wk)	Administration (4 wk)	Follow-up (6 wk)
Age (y)		25.5 ± 6.4			26.0 ± 8.0		
Male		8			9		
Female		19			16		
Weight	(kg)	59.4 ± 9.4	59.0 ± 9.2	59.2 ± 9.5	58.2 ± 8.9	58.0 ± 8.9	57.8 ± 9.0
BMI	(kg/m^2^)	21.9 ± 2.2	21.7 ± 2.1	21.8 ± 2.2	21.6 ± 2.6	21.6 ± 2.6	21.5 ± 2.7
Body fat	(%)	24.0 ± 3.5	24.0 ± 3.3	24.0 ± 3.5	23.2 ± 3.9	23.1 ± 4.4	23.9 ± 4.0
Height	(cm)	164.1 ± 7.5			163.4 ± 5.8		
WC		71.2 ± 5.8	70.5 ± 7.4	70.6 ± 7.3	70.1 ± 8.0	69.7 ± 8.2	69.6 ± 8.2
RC		93.4 ± 5.1	92.9 ± 4.9	93.4 ± 5.0	91.4 ± 5.4	122.0 ± 57.8	91.5 ± 4.8
MAC		25.5 ± 2.8	25.2 ± 2.9	25.1 ± 2.8	24.6 ± 2.6	24.3 ± 2.5	24.5 ± 2.4
TSF		20.3 ± 4.1	20.3 ± 3.6	20.1 ± 3.5	18.7 ± 3.3	19.0 ± 3.1	18.4 ± 2.8
SBP	(mmHg)	110.1 ± 9.2	110.2 ± 8.9	107.8 ± 7.6	107.8 ± 8.8	107.6 ± 7.5	106.8 ± 9.9
DBP		70.2 ± 6.3	68.9 ± 6.7	68.7 ± 5.8	70.2 ± 7.1	69.4 ± 6.2	69.1 ± 7.4
HR	(bpm)	74.3 ± 5.1	73.7 ± 6.4	75.3 ± 7.3	75.8 ± 7.2	75.4 ± 7.2	75.3 ± 6.8

BMI: body weight (kg)/[body height]^2^ (m^2^); WC: waist circumference; RC: rump circumference; MAC: midarm circumference; TSF: triceps skinfold; SBP: systolic blood pressure; DBP: diastolic blood pressure; HR: heart rate. Student's *t*-test, no significant difference between placebo and treatment groups at week 0. Values are mean ± SD (treatment group: n = 27; placebo group: n = 25).

### 3.5 Safety tests (biochemical blood markers) (clinical trial II)

Examination of blood lipid changes (Table 3) revealed no significant differences in the total cholesterol (TC), triglycerides (TG), low-density lipoprotein cholesterol (LDL-C), and high-density lipoprotein cholesterol (HDL-C) in subjects in the treatment and placebo groups after 4 weeks. Examination of blood glucose revealed no significant changes in fasting blood glucose (FBG) and glycated hemoglobin (HbA1c) levels in the treatment group or placebo group (Table 3). Markers of liver, kidney, and heart function were within normal values before and after administration of the Vigiis 101-LAB capsule II or the placebo, and there were no significant changes (Table 3). Thus, the Vigiis 101-LAB capsule II does not cause side effects in the liver, kidney, serum creatine concentration, uric acid, or heart function. Measurement of blood counts and *in vivo* electrolytes revealed no significant changes at 4 weeks after administration of the Vigiis 101-LAB capsule II. In this trial, there were no safety concerns with the administration of the Vigiis 101-LAB capsule II or the placebo.

**Table 3:** Effect of chronic administration of Vigiis 101-LAB capsule II or placebo on blood lipid, blood glucose balance, liver, kidney function, and electrolyte balance profiles of subjects (clinical trial II).

		Treatment group			Placebo group		
		Initial (0 wk)	Administration (4 wk)	Follow-up (6 wk)	Initial (0 wk)	Administration (4 wk)	Follow-up (6 wk)
TC	(mg/dL)	171.1 ± 24.6	172.9 ± 24.7	170.3 ± 22.8	167.6 ± 21.0	172.5 ± 19.5	173.3 ± 23.1
TG		72.6 ± 29.2	76.9 ± 30.5	75.8 ± 33.5	76.4 ± 39.3	72.9 ± 25.6	85.3 ± 38.6
LDL-C		96.2 ± 21.6	100.8 ± 25.0	98.4 ± 23.6	89.8 ± 20.4	94.0 ± 18.0	93.4 ± 21.4
HDL-C		55.8 ± 7.7	54.6 ± 8.8	52.7 ± 8.8	59.1 ± 12.2	60.5 ± 11.5	58.8 ± 12.0
FBG		88.9 ± 5.6	87.9 ± 5.2	88.1 ± 4.9	87.6 ± 4.6	86.2 ± 4.0	84.0 ± 5.7
HbA1c	(%)	5.2 ± 0.2	5.2 ± 0.2	5.2 ± 0.2	5.2 ± 0.2	5.1 ± 0.2	5.2 ± 0.2
AST	(IU/L)	19.2 ± 2.6	17.8 ± 2.6	18.4 ± 2.5	20.5 ± 3.5	19.9 ± 3.6	20.9 ± 4.1
ALT		17.0 ± 3.9	16.3 ± 3.8	17.4 ± 4.0	19.1 ± 7.6	18.0 ± 7.2	20.0 ± 7.8
γ-GT		11.7 ± 3.0	12.2 ± 3.0	11.9 ± 2.7	16.8 ± 10.4	18.8 ± 12.4	19.0 ± 12.5
Albumin	(g/dL)	4.4 ± 0.2	4.6 ± 0.2	4.5 ± 0.2	4.3 ± 0.2	4.5 ± 0.2	4.5 ± 0.21
Total-protein		7.1 ± 0.3	7.2 ± 0.3	7.2 ± 0.3	7.0 ± 0.3	7.1 ± 0.3	7.2 ± 0.23
BUN	(mg/dL)	10.8 ± 2.4	9.6 ± 2.3	10.6 ± 1.8	10.8 ± 2.3	10.4 ± 2.3	11.4 ± 2.6
UA		5.1 ± 0.9	5.0 ± 0.8	5.2 ± 0.8	4.9 ± 0.7	4.9 ± 0.8	5.1 ± 0.7
Creatinine		0.8 ± 0.1	0.8 ± 0.1	0.8 ± 0.1	0.8 ± 0.2	0.8 ± 0.2	0.8 ± 0.2
Ca	(mg/dL)	9.4 ± 0.2	9.7 ± 0.3	9.6 ± 0.3	9.4 ± 0.2	9.5 ± 0.2	9.6 ± 0.2
P		4.1 ± 0.4	4.2 ± 0.4	4.3 ± 0.5	4.2 ± 0.4	4.2 ± 0.4	4.1 ± 0.4
Mg		2.1 ± 0.1	2.1 ± 0.1	2.1 ± 0.1	2.1 ± 0.1	2.1 ± 0.1	2.1 ± 0.1
N	(mmol/L)	138.7 ± 1.6	139.6 ± 1.3	138.6 ± 0.9	138.4 ± 1.3	139.0 ± 1.2	138.4 ± 1.3
K		4.1 ± 0.2	4.1 ± 0.1	4.1 ± 0.2	4.1 ± 0.2	4.1 ± 0.2	4.1 ± 0.2
Cl		105.2 ± 1.4	103.9 ± 1.3	104.0 ± 1.3	105.6 ± 1.8	104.3 ± 1.4	103.8 ± 1.5
Fe	(μg/dL)	86.2 ± 25.6	88.7 ± 31.9	70.9 ± 27.0	73.3 ± 21.6	81.6 ± 22.1	86.2 ± 32.1
CPK	(IU/L)	102.3 ± 39.4	85.1 ± 27.0	90.7 ± 29.6	93.4 ± 32.5	91.0 ± 38.3	86.1 ± 38.4

TC: total cholesterol; TG: triglyceride; LDL-C: low-density lipoprotein cholesterol; HDL-C: high-density lipoprotein cholesterol; FBG: fasting blood glucose; HbA1c: glycated hemoglobin; AST, aspartate aminotransferase; ALT, alanine aminotransferase; γ-GTP, γ-glutamyl transpeptidase; BUN, blood urea nitrogen. UA: uric acid. CPK: creatinine phosphokinase. Student's *t*-test, no significant difference between placebo and treatment groups at week 0. Values are mean ± SD (treatment group: n = 27; placebo group: n = 25).

### 3.6 Changes in inflammation markers and antioxidant activity (clinical trial II)

For inflammation markers, immunoglobulin G (IgG) and immunoglobulin M (IgM) levels were significantly increased in subjects in the test group (Table 4). However, there were no significant changes in high-sensitivity C-reactive protein (Hs-CRP) and immunoglobulin E (IgE), which were within the normal range before and after administration. In the placebo group, IgG, IgM, Hs-CRP, and IgE levels showed no significant changes, which were maintained within the normal range before and after the intervention. For antioxidant activity, TEAC was significantly increased from 6.6 ± 0.04 to 6.7 ± 0.05 (μg Trolox eq./mg) after subjects were administered NTU 101 probiotics. Reactive oxygen species (ROS) serve important physiological functions; thus, inappropriate removal of ROS may cause paradoxical reductive stress and thereby induce or promote disease (Schmidt et al., 2015). As molecules in the body scavenge excessive free radicals, an increase in TEAC under normal physiological conditions indicates that the ability to scavenge free radicals is enhanced (Arnao et al., 1996; Hodges et al., 1999; Yu et al., 2016). GSH was significantly increased from 31.6 ± 2.6 to 33.0 ± 4.8. GSH acts as the first line of antioxidant defense in the human body. The physiological function of GSH is conferred by the sulfhydryl (-SH groups) present in GSH molecules. When these groups encounter free radicals, they tend to release hydrogen ions to form sulfide free radicals, thereby scavenging free radicals and increasing antioxidant capacity (Sheng et al., 2016). TBARS were significantly decreased from 0.71 ± 0.17 to 0.43 ± 0.09. Lipid peroxidation typically involves a series of free radical reactions alters the lipid composition of the cell membrane, causing denaturation of intramembrane enzymes and proteins and changes in cell structure and function. Therefore, scavenging harmful free radicals and terminating lipid peroxidation can effectively prevent disease (Lobo et al., 2010). No significant differences were observed in terms of these three items in the placebo group. These results showed that consuming NTU 101 probiotics significantly increases TEAC and GSH levels, thereby significantly decreasing the synthesis of lipid peroxides in the plasma. For erythrocyte enzyme activity, GSH Px was significantly increased from 86.2 ± 16.0 to 135.8 ± 36.6 after NTU 101 probiotics were administered. GSH Px is a major intracellular, water-soluble antioxidant that protects cells from free radicals. GSH Px uses GSH as a reducing agent and selenium as a cofactor to reduce its substrates to stable alcohols (R-O-H), thereby increasing antioxidant capacity and reducing damage caused by free radicals (Kleniewska et al., 2016). The activity of GSH Rd was also significantly increased from 42.2 ± 0.2 to 49.9 ± 0.4. GSH Rd reduces oxidized GSH to reduced GSH using NADPH as a source of hydrogen. This enables the scavenging of free radicals and increases antioxidant capacity (Bridgman et al., 2017). These showed no significant differences in the placebo group. GSH Px and GSH Rd constitute a dynamic equilibrium and form an effective antioxidant system. These results showed that NTU 101 probiotics increase the activity of GSH Px and GSH Rd in erythrocytes.

**Table 4:** Effect of chronic administration of Vigiis 101-LAB capsule II or placebo on immunoassay, antioxidant capacity, and the GSH redox cycle profiles of subjects (clinical trial II).

		Treatment group			Placebo group		
		Initial (0 wk)	Administration (4 wk)	Follow-up (6 wk)	Initial (0 wk)	Administration (4 wk)	Follow-up (6 wk)
Hs-CRP	(mg/dL)	0.09 ± 0.09	0.08 ± 0.06	0.09 ± 0.09	0.18 ± 0.24	0.09 ± 0.10	0.14 ± 0.16
IgG		1190.3 ± 180.5	1230.6 ± 178.4∗	1215.2 ± 176.8∗	1217.7 ± 160.0	1260.6 ± 184.6	1275.8 ± 159.4
IgM		132.1 ± 36.3	141.0 ± 41.5∗	134.1 ± 40.6	123.3 ± 42.7	128.4 ± 45.7	128.1 ± 44.7
IgE	(IU/mL)	83.6 ± 173.1	84.0 ± 73.7	89.1 ± 79.3∗	109.2 ± 94.2	118.1 ± 106.4	120.3 ± 106.7
GSH	(ng/μL)	31.6 ± 2.6	33.0 ± 4.8∗	37.0 ± 2.0∗	29.5 ± 3.0	30.2 ± 2.6	31.4 ± 1.6
GSH Px	(mU/mL)	86.2 ± 16.0	135.8 ± 36.6∗	113.5 ± 12.3∗	88.9 ± 17.6	94.3 ± 7.3	95.6 ± 24.0
GSH Rd		42.2 ± 0.2	49.9 ± 0.4∗	48.2 ± 1.9∗	42.5 ± 0.3	42.7 ± 0.4	42.6 ± 0.2
TEAC	(μg Trolox eq./mg)	6.60 ± 0.04	6.70 ± 0.05∗	6.70 ± 0.07∗	6.60 ± 0.06	6.60 ± 0.06	6.60 ± 0.06
TBARS	(μM)	0.71 ± 0.17	0.43 ± 0.09∗	0.32 ± 0.07∗	0.51 ± 0.14	0.68 ± 0.30	0.51 ± 0.12

		Treatment			Placebo		
		Initial	Week 4	p-value	Initial	Week 4	p-value

Gut transit times		107.2 ± 29.3 min	80.6 ± 33.7∗min	0.004	82.8 ± 48.9 min	99.0 ± 34.1 min	0.052


Hs-CRP: high-sensitivity C-reactive protein; IgG: immunoglobulin G; IgM: immunoglobulin M; IgE: Immunoglobulin E; GSH: glutathione; GSH Px: glutathione peroxidase; GSH Rd: glutathione reductase; TEAC: Trolox equivalent antioxidant capacity; TBARs: thiobarbituric acid reactive substances. Student's *t*-test, no significant difference between placebo and treatment groups at week 0. Values are mean ± SD (treatment group: n = 27; placebo group: n = 25). ∗ Significantly different from the initial value (P < 0.05).

### 3.7 Changes in chyme passage time through the gut (clinical trial II)

The results from week 4 of the NTU 101 probiotic intervention showed that the gut passage time of chyme was significantly shortened from 107.2 ± 29.3 to 80.6 ± 33.7 min, while no significant differences were observed after intervention in subjects in the placebo group (Table 4).

## 4 Discussion

The length of the human gastrointestinal tract is 6–9 m. The gut is the largest immune organ in the human body, and gut-associated lymphoid tissues are an essential component of the body's immune system that acts as the first line of immune defense. Gut flora is relatively complex as it contains at least 300 types of bacteria that number up to one hundred trillion. Maintaining the gut flora balance enables the body to maintain a healthy state. Major changes have occurred in the gut flora of modern people as their diet has become increasingly refined. This has gradually reduced the levels of beneficial bacteria and increased harmful bacteria. This causes people to be prone to fatigue, anxiety, and skin roughness, affects nutrient absorption, and causes disease. Metabolites in the gut flora have regulatory effects on the immune system (Lei et al., 2016; Sun & O'Riordan, 2013; Schuijt et al., 2013).

Clinical trial I adopted a randomized, double-blind design, in which subjects were randomized into test and placebo groups. Subjects in the test group were administered Vigiis 101-LAB capsules I every day, and changes in the gut flora were examined regularly each week during the trial. As the study progressed, the count numbers of *Bifidobacteria* and *Lactobacillus* in the feces of the test group significantly increased compared to that in the placebo group (Table 1). There were no significant differences in the *C. perfringens,* or *E. coli* counts in the feces of the subjects from the test and placebo groups after 4 weeks of test product administration (both *P* > 0.05) (Table 1). No major differences in stool morphology were observed in participants in the test group before and after the trial. Thus, this test product does not cause constipation or diarrhea. The Vigiis 101-LAB *Lactobacillus* powder increased *Bifidobacteria* and *Lactobacillus* counts in human feces, improved the ratio of beneficial to harmful bacteria in the gut, and played a role in increasing the proliferation of beneficial bacteria in the body, thereby improving gut flora.

The results of the clinical studies showed that probiotics could alter the composition of gut flora to help humans resist various pathogens and inhibit harmful bacteria, restore the balance of gut flora, and enhance the defensive capability of the digestive tract. Studies have shown that, in addition to its effects on host digestive function, gut flora also affects other physiological functions, particularly the body's immune system (Hooper et al., 2012; Bengmark, 2013; Atarashi et al., 2011; Thursby and Juge, 2017). The digestive system contains many epithelial cell loop folds and villi to facilitate food absorption and immunity. The mechanisms by which *Lactobacillus* attaches to the gut are unclear. Some researchers have suggested that bacteria attach to the gut via lectins on the bacteria surface that can interact with mannose on epithelial cells (Krasowska and Sigler, 2014). Other researchers suggested that adherence occurs because of the production of S-layer proteins. They indicated that adherence is related to the hydrophobicity of peptidoglycan on bacterial cell walls (Tuson and Weibel, 2013). Greater hydrophobicity makes it easier for bacteria to adhere (Stecher, 2015). The harmful bacterium *Clostridium difficile* adheres to Caco-2 cells and secretes toxins that cause cell damage. Supplementation with the probiotic *Lactobacillus rhamnosus* GG inhibits the growth of *C. difficile* and competes for adherence, thereby protecting the gut from pseudomembranous colitis caused by *C. difficile*. Gut flora can also compete for nutrients and regulate host immune responses to resist colonization by foreign and intrinsic pathogens (Kamada et al., 2013; Belkaid and Hand, 2014).

Gut flora can affect the immune system, in particular the development of gut-associated lymphoid tissue. For instance, isolated lymphoid follicles from gut-associated lymphoid tissue in germ-free mice cannot mature, and the number of lymphocytes on the gut epithelium of these mice secreting IgA and CD8αβ is impaired. Additionally, cytokines induced by gut flora or the Toll-like receptor immune response are crucial factors in immune pathways. These cytokines can enter relevant functional regions inside the brain by free diffusion or transporters in the blood-brain barrier and induce a series of effects on the central nervous system. The gut flora also has significant effects on the growth and development of immune cells (Belkaid and Harrison, 2017; Tomkovich and Jobin, 2016; Agace and McCoy, 2017). Studies showed that the number of regulatory T-cells in the colonic lamina propria of germ-free mice was significantly reduced. Thus, the development of peripherally induced regulatory T-cells depends on the presence of gut flora (Smith and Garrett, 2011). Another study found that the number of T-helper 17 cells in germ-free mice treated with antibiotics was significantly reduced and that segmented filamentous bacteria induced the synthesis and differentiation of T-helper 17 cells (Lee and Kim, 2017).

Gut health is important to the entire digestive system and an essential component of the immune system. The adherence and non-adherence of probiotic *Lactobacillus* stimulate immunoregulation and affect the inhibition of harmful bacteria and nutrient absorption efficiency. Therefore, both gastroenterology experts and nutritionists have continuously emphasized the vital correlation between the gut and human health in recent years (Pandey et al., 2015). The main aim of probiotic foods is to create a gut ecosystem that tips the balance between beneficial and harmful bacteria. Probiotics are live bacteria that promote changes in the gut flora balance in the host. Probiotics are selected based on whether they resist damage under conditions of strong acidity and high concentrations of bile salts in the gut, can adhere to the gut mucosa of the host, and can benefit host physiological functions (Ruiz et al., 2013; Shehata et al., 2016).

The antioxidant systems in the human body consist of powerful non-enzymatic and enzymatic antioxidants (Halliwell, 2007). The antioxidant enzymes in all body cells consist of three major classes of antioxidant enzymes which are the catalases, superoxide dismutases, and glutathione peroxidases (GPX), all of these, play crucial roles in maintaining homeostasis into cells (Cheung et al., 2001). The role of GPx is achieved by the reduction of hydrogen peroxide, lipid hydroperoxides and other organic hydroperoxides (Tappel et al., 1982). Glutathione-S-transferases (GST) represent a major group of detoxifying enzymes (Hayes and Pulford, 1995), which form a family of multifunctional proteins involved in the cellular detoxification of cytotoxic and genotoxic compounds and in the protection of tissues against oxidative damage (Mannervik and Danielson, 1988; Pickett and Lu, 1989). Glutathione system includes glutathione S-transferases, glutathione peroxidases, and glutathione reductase. These enzymes help in detoxification mechanism (Hayes et al., 2005). Glutathione reductase (GR) catalyzes the reduction of oxidized glutathione (GSSG) to reduced glutathione (GSH). This enzyme enables the cell to sustain adequate levels of cellular GSH.

The results of the clinical trial presented in this report showed that administration of Vigiis 101-LAB probiotics could increase the proliferation of beneficial bacteria in the body, thereby improving gut flora. Additionally, IgG, IgM, TEAC, GSH, GSH Px, and GSH Rd activities were all significantly increased, while TBARS and chyme passage time through the gut were significantly reduced. Thus, the Vigiis 101-LAB capsule II administration significantly improves gut flora, peristalsis, immunity, and anti-oxidative capacity.

## 5 Conclusion

In clinical trial I, we examined the effects of Vigiis 101-LAB capsules I (5 billion CFU/day) on improving gut microflora. After administration for 4 weeks, *Bifidobacterium* spp. and *Lactobacillus* spp. counts were significantly higher in the feces of the experimental group than that of the placebo group, with increases of 4.01- and 4.25-fold, respectively.

In clinical trial II, peristalsis, immunity, and anti-oxidative capacity were investigated, after administration for 4 weeks of Vigiis 101-LAB capsule II (10 billion CFU/day). The results showed improved gut motility, decreased food transit time, and significantly increased antibody IgG, IgM, and antioxidant activity in the experimental group compared with that of the placebo group. Thus, administering Vigiis 101 capsule II is considered to have health benefits of improved peristalsis, immunity, and anti-oxidative capacity.

## Declarations

### Author contribution statement

Chien-Li Chen, Jyh-Ming Liou: Conceived and designed the experiments; Analyzed and interpreted the data; Wrote the paper.

Tsong-Ming Lu, Yi-Hsien Lin: Performed the experiments; Analyzed and interpreted the data.

Chin-Kun Wang: Conceived and designed the experiments; Analyzed and interpreted the data.

Tzu-Ming Pan: Conceived and designed the experiments; Analyzed and interpreted the data; Contributed reagents, materials, analysis tools or data.

### Funding statement

This work was supported by SunWay Biotech Co., Ltd., Taipei, Taiwan.

### Competing interest statement

The authors declare no conflict of interest.

### Additional information

No additional information is available for this paper.
